# 

**Published:** 1908-02-08

**Authors:** 


### CONTENTS.

| | PAGE |
|---|---|
| **Unqualified Practice, the Medical Profession and the Public** | 487 |
| **Transmission of Acquired Characteristics** | 488 |
| **Annotations—** Kissing the Book—Syphilis in the Third Generation—Subcutaneous Fibrous Nodules | 489 |
| **Medical Opinion and Movement** | 490 |
| **Hospital Clinics—** Oral Sepsis and its Relation to Abdominal Disease. By John H. Dauber, M.A., M.B., B.Ch., Oxon. | 491 |
| **Special Article—** The Treatment of Periosteal Sarcoma | 494 |
| **Medicine—** Acute Gout | 495 |
| A Case for Diagnosis | 496 |
| **Points in Surgery—** Psoas Abscess.—II. | 497 |
| Some Diseases of the Male Genital System | 498 |
| **The Royal Army Medical Corps Section—** The Training of Officers | 499 |
| **Resident Medical Officers' Department—** House Appointments at Small Hospitals | 500 |
| **Public Health and Hygiene—** Should Consumptives be Segregated? | 501 |
| **Pathology in General Practice—** The Examination of the Urine for Sugar | 502 |
| **Therapeutics—** Prescriptions and Dispensing | 503 |
| Secret Remedies and Proprietary Products.—II. | 504 |
| **Hospital Administration—** The London Hospital | 505 |
| Social and Poor-law Problems | 506 |
| Tonbridge Cottage Hospital | 508 |
| News and Coming Events | 509 |
| The Graduates' Medical Schools | 511 |
| **Nursing Administration—** The Poor-law Midwifery Schools | 510 |
| The Common Task | 511 |
| **Editor's Letter-Box—** The Mission to Lepers in India and the East | 511 |
| The North-Eastern Hospital for Children | 512 |

